# Crystal structure of an ep­oxy­sterol: 9α,11α-ep­oxy-5α-cholest-7-ene-3β,5,6α-triol 3,6-di­acetate

**DOI:** 10.1107/S2056989017013391

**Published:** 2017-10-06

**Authors:** Vincenzo Piccialli, Angela Tuzi, Roberto Centore

**Affiliations:** a Dipartimento di Scienze Chimiche, Università degli Studi di Napoli ’Federico II’, Complesso di Monte S. Angelo, Via Cinthia, 80126 Napoli, Italy

**Keywords:** crystal structure, steroids, hydrogen bond

## Abstract

The title compound, C_31_H_48_O_6_, is a polyoxygenated ep­oxy steroid obtained by a multi-step synthesis involving oxidation of 7-de­hydro­cholesterol. It crystallizes in the *P*2_1_2_1_2_1_ space group; however, the absolute structure of the molecule in the crystal could not be determined by resonant scattering. The configuration at the C5 and C6 positions is in both cases of the α-type, as is that of the C atoms of the ep­oxy ring. Mol­ecules in the crystal form chains parallel to the *b* axis by hydrogen bonding between O—H donors and carbonyl O-atom acceptors. Some atoms of the alkyl chain are disordered over two orientations, with a refined occupancy ratio of 0.511 (10):0.489 (10).

## Abstract 2

The title compound is a polyoxygenated ep­oxy steroid that crystallizes in the *P*2_1_2_1_2_1_ space group.

## Chemical context   

**Figure 1:**
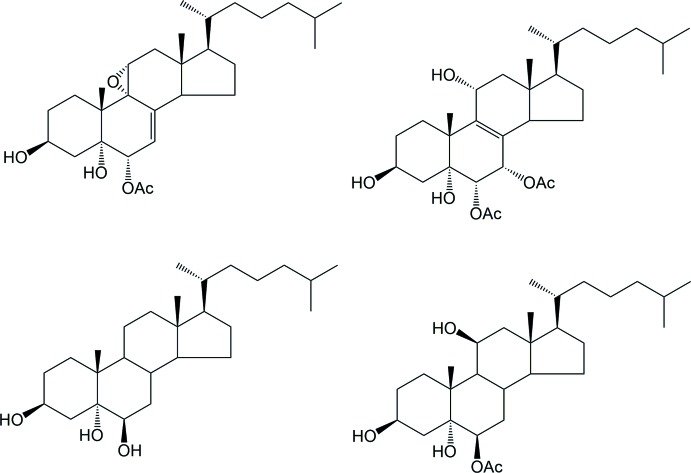
Selected biologically active polyoxygenated steroids of marine origin.

Polyoxygenated steroids (Fig. 1 ▸) are metabolites both of terrestrial and marine origin possessing a number of remarkable biological activities (D’Auria *et al.*, 1993 ▸). Our previous studies in this field focused on the isolation and synthesis of a number of such substances possessing new nuclear oxygenation patterns (Madaio *et al.*, 1988 ▸; Migliuolo *et al.*, 1992 ▸). In this context, new ruthenium tetroxide-catalysed oxidation methods (Bifulco *et al.*, 2003*a* ▸,*b* ▸; Piccialli *et al.*, 2007 ▸, 2010 ▸; Piccialli, 2014 ▸) were developed to introduce suitable oxygenated functionalities in the *B*, *C* and *D* rings of the steroid nucleus. Among others, 9,11-ep­oxy­sterols have been isolated from various marine organisms (Gunasekera *et al.*, 1983 ▸) and display diverse biological activities. In particular, the 3-deacetyl analogue of the title compound (Fig. 1 ▸) has shown to inhibit the binding of [I125] IL-8 to the human recombinant IL-8 receptor type A (de Almeida Leone *et al.*, 2000 ▸).

**Figure 2:**
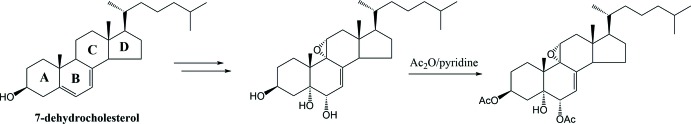
Synthesis of the title compound.

We are carrying out a broad research program aimed at discovering new biologically active substances. In recent years, we have synthesized and studied, among others, purine nucleoside analogues (D’Errico *et al.*, 2011 ▸, 2012*a* ▸,*b* ▸; D’Atri *et al.*, 2012 ▸; Oliviero *et al.*, 2008 ▸, 2010*a* ▸,*b* ▸), cyclic ethers and polyethers (Piccialli *et al.*, 2007 ▸, 2009 ▸; Piccialli, D’Errico *et al.*, 2013 ▸; Piccialli, 2014 ▸) and nitro­gen-rich fused-ring compounds (Centore *et al.*, 2013 ▸). Within this program, and on the basis of the reduced amount of direct structural information available on ep­oxy steroids, we have synthesized the title compound (**1**), by di­acetyl­ation of **3**, in turn obtained from cheap commercially available 7-de­hydro­cholesterol (**2**) (see Fig. 2 ▸), according to a previously reported procedure (Migliuolo *et al.*, 1991 ▸). In particular, during the synthesis, two diastereomers, with opposite configuration at C6, were obtained, with predominance of the *trans*-isomer (5α-OH/6β-OH). The structural analysis was performed in order to unambiguously assign the configuration of the title compound.
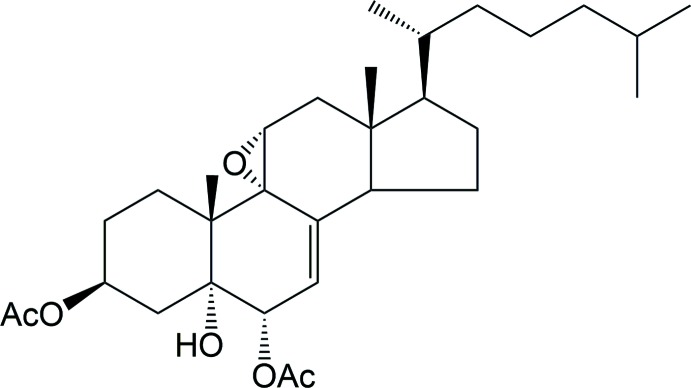


## Structural commentary   

**Figure 3:**
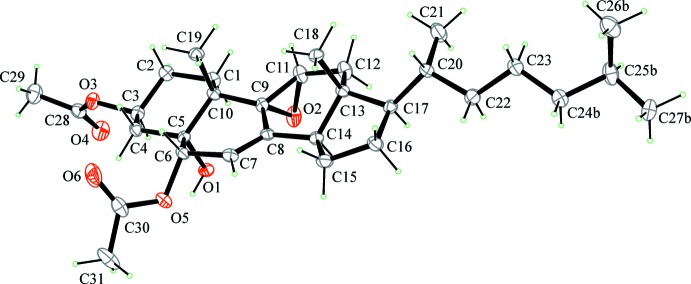
*ORTEP* view of the mol­ecular structure of the title compound. Displacement ellipsoids are drawn at 30% probability level. Only the most populated orientation of the disordered chain is shown.

The crystallographically independent mol­ecule is shown in Fig. 3 ▸. From the figure it is evident that the two acet­yloxy groups have a different stereochemical orientation (3β,6α) and that the stereochemical orientation of the hy­droxy group is the same as that of the acet­yloxy group at C6 (5α,6α). In addition, the orientation of the ep­oxy oxygen atom is on the opposite side as compared with the methyl groups C18 and C19 (9α,11α-ep­oxy) and on the same side of the hy­droxy group bonded to C5. The stereoselectivity in the formation of the ep­oxy ring is probably related to the steric hindrance due to the methyl groups.

**Figure 4:**
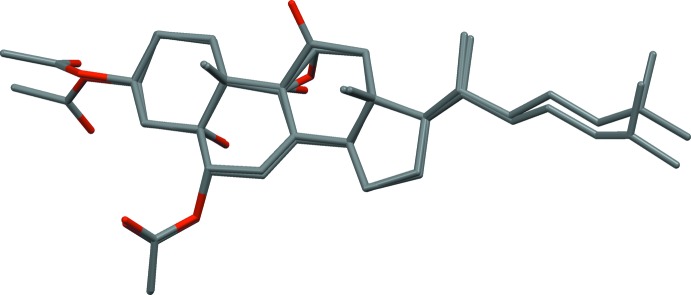
Overlay of the X-ray mol­ecular structure of the title compound with the previously reported 3β,6α-diacet­oxy-5,9α-dihy­droxy-5α-cholest-7-en-11-one (Piccialli, Tuzi *et al.*, 2013 ▸).

We have reported the crystal structure of a steroid closely related to the title compound (Piccialli, Tuzi *et al.*, 2013 ▸), in which the two acet­yloxy groups, the C18 and C19 methyl groups and the alkyl tail have the same configuration as in the present one, and, moreover, an α hy­droxy group at C9 and a keto group at C11 are present. In Fig. 4 ▸ the two mol­ecular structures are superimposed. The superposition is very good, apart for a small difference in the torsion angle for the acetyl group at C3.

## Supra­molecular features   

**Figure 5:**
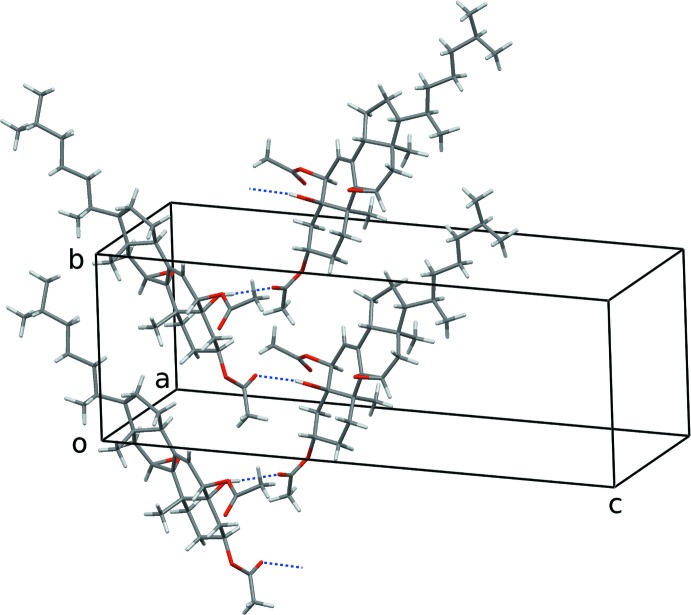
Partial crystal packing of the title compound. Only the most populated orientation of the disordered chain is shown.

The crystal packing of the title compound is shown in Fig. 5 ▸. Mol­ecules in the crystal form chains by hydrogen bonding between the alcohol O1—H donor and the O4 carbonyl acceptor (Table 1 ▸). The chains run parallel to the *b* axis and are wrapped around a 2_1_ crystallographic screw axes. Adjacent chains along the *a* axis are held by weak hydrogen bonding between C29—H donor and O6 carbonyl acceptor.

**Figure 6:**
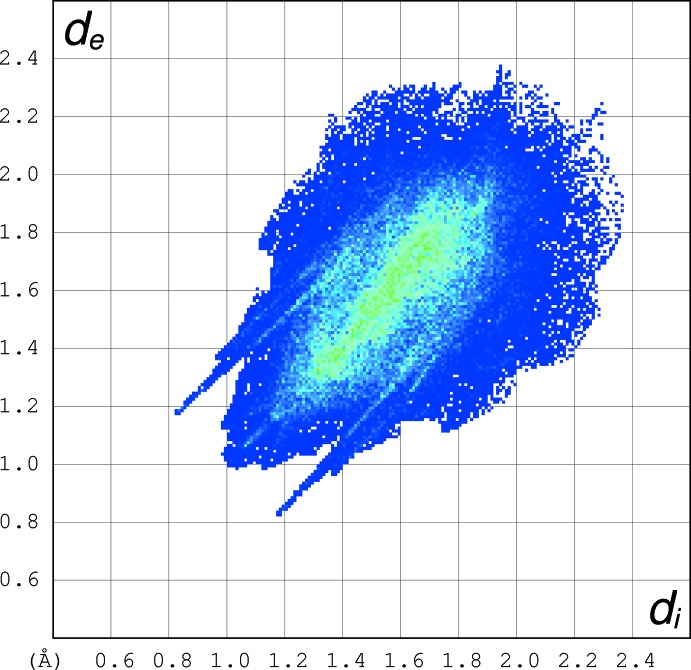
Hirshfeld fingerprint plot of the crystallographically independent mol­ecule of the title compound.

In order to detect additional packing features, we have examined the Hirshfeld surface (Spackman & McKinnon, 2002 ▸; Wolff *et al.*, 2012 ▸). In Fig. 6 ▸ the Hirshfeld fingerprint plot of the independent mol­ecule is reported. In the plot, for each point of the Hirshfeld surface enveloping the mol­ecule in the crystal, the distance *d*_i_ to the nearest atom inside the surface and the distance *d*_e_ to the nearest atom outside the surface are shown. The color of each point in the plot is related to the abundance of that inter­action, from blue (low) to green (high) to red (very high).

A distinctive feature of the plot is represented by the two blue spikes at *d*_i_ + *d*_e_ = 2.0 Å, pointing to the lower left of the plot and symmetrically disposed with respect to the diagonal. They correspond to the strong hydrogen bonds present in the packing. Another feature is the central green strip along the diagonal, centered at *d*_i_ + *d*_e_ = 3.2 Å, indicating a large number of loose H⋯H contacts. As expected, they are the predominant inter­molecular contacts in the packing of the title compound. The central green strip ends up in the blue sting at at *d*_i_ = *d*_e_ = 1.0 Å, which reflects points on the Hirshfeld surface that involve nearly head-to-head close H⋯H contacts.

## Database survey   

A search of the Cambridge Structural Database (CSD version 5.38, last update February 2017; Groom *et al.*, 2016 ▸) gave no match for the title compound. We have searched, within steroids with a double bond at C7 (122 hits in total), for an additional ep­oxy group in any of the *A*, *B*, *C* and *D* rings of the steroid moiety. We found eight hits, with the following refcodes and position of the ep­oxy ring: EZELAX (4β,5β-ep­oxy), DIZPUY and FIWYUG (9α,11α-ep­oxy), RUGDIH (9α,13α-ep­oxy), POHDEW (9α,14α-ep­oxy), QULRAS and QULRIA (13α,17α-ep­oxy), BEXCHO (14α,15α-ep­oxy).

## Synthesis and crystallization   

5α-Cholest-7-ene-3β,5,6α-triol 3,6-di­acetate was obtained from 7-de­hydro­cholesterol as described (Fieser *et al.*, 1953 ▸; Migliuolo *et al.*, 1991 ▸), followed by acetyl­ation. Mercuric acetate de­hydrogenation gave the Δ^7,9(11)^-analogue. Hydrolytic de­acetyl­ation, MnO_2_ oxidation at C6 and subsequent *meta*-chloro­perbenzoic acid epoxidation at the C9—C11 double bond gave 9α,11α-ep­oxy-3β,5-dihy­droxy-5α-cholest-7-en-6-one. LiAlH_4_ reduction of the C6 ketone function in the latter, followed by acetyl­ation with Ac_2_O/py, furnished the title compound **1** and its C6 epimer, in a 1:4 ratio. The pure title compound was obtained by HPLC separation (CHCl_3_/MeOH, 96:4 *v*/*v*). The compound was dissolved in a minimal amount of CHCl_3_ and the solution was left to evaporate slowly at room temperature to give crystals suitable for X-ray diffraction analysis.

## Refinement   

Crystal data, data collection and structure refinement details are summarized in Table 2 ▸. The H atoms were generated stereochemically and were refined by the riding model. The alcohol H atom was refined freely with *U*_iso_(H) =1.2*U*_eq_(O). All other H atoms were refined with *U*_iso_(H) = 1.2*U*_eq_(C) or 1.5*U*_eq_(C) for methyl H atoms. A rotating model was used for most methyl groups. The C25 and C26 atoms of the alkyl chain are disordered over two orientations. The two split positions were refined by applying DFIX and SAME restraints on bond lengths. The final refined occupancy factors of the two components of disorder are 0.511 (10) and 0.489 (10).

## Supplementary Material

Crystal structure: contains datablock(s) global, I. DOI: 10.1107/S2056989017013391/rz5221sup1.cif

Structure factors: contains datablock(s) I. DOI: 10.1107/S2056989017013391/rz5221Isup2.hkl

Supporting information file. DOI: 10.1107/S2056989017013391/rz5221Isup3.cml

CCDC reference: 1575390

Additional supporting information:  crystallographic information; 3D view; checkCIF report

## Figures and Tables

**Table 1:** Hydrogen-bond geometry (Å, °)

*D*—H⋯*A*	*D*—H	H⋯*A*	*D*⋯*A*	*D*—H⋯*A*
C29—H29*B*⋯O6^i^	0.98	2.61	3.567 (6)	166
O1—H1*O*⋯O4^ii^	0.81 (3)	2.16 (4)	2.923 (3)	158 (4)

Symmetry codes: (i) 
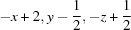
; (ii) 
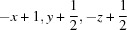
.

**Table 2:** Experimental details

Crystal data	
Chemical formula	C_31_H_48_O_6_
*M*_r_	516.69
Crystal system, space group	Orthorhombic, *P*2_1_2_1_2_1_
Temperature (K)	173
*a*, *b*, *c* (Å)	9.8990 (13), 10.1030 (16), 28.961 (6)
*V* (Å^3^)	2896.4 (8)
*Z*	4
Radiation type	Mo *K*α
μ (mm^−1^)	0.08
Crystal size (mm)	0.50 × 0.30 × 0.10

Data collection	
Diffractometer	Bruker–Nonius KappaCCD
Absorption correction	Multi-scan (*SADABS*; Bruker, 2001 ▸)
*T*_min_, *T*_max_	0.949, 0.980
No. of measured, independent and observed [*I* > 2σ(*I*)] reflections	14033, 5983, 3810
*R*_int_	0.060
(sin θ/λ)_max_ (Å^−1^)	0.650

Refinement	
*R*[*F* ^2^ > 2σ(*F* ^2^)], *wR*(*F* ^2^), *S*	0.058, 0.121, 1.02
No. of reflections	5983
No. of parameters	364
No. of restraints	9
H-atom treatment	H atoms treated by a mixture of independent and constrained refinement
Δρ_max_, Δρ_min_ (e Å^−3^)	0.19, −0.19
Absolute structure	Flack *x* determined using 3518 quotients [(*I* ^+^)−(*I* ^−^)]/[(*I* ^+^)+(*I* ^−^)] (Parsons *et al.*, 2013 ▸)
Absolute structure parameter	−1.6 (8)

Computer programs: *COLLECT* (Nonius, 1999 ▸), *DIRAX/LSQ* (Duisenberg *et al.*, 2000 ▸), *EVALCCD* (Duisenberg *et al.*, 2003 ▸), *SIR97* (Altomare *et al.*, 1999 ▸), *SHELXL2016* (Sheldrick, 2015 ▸), *ORTEP-3 for Windows* and *WinGX* (Farrugia, 2012 ▸) and *Mercury* (Macrae *et al.*, 2006 ▸).

## supplementary crystallographic information

### Crystal data

**Table:**

C_31_H_48_O_6_	*D*_x_ = 1.185 Mg m^−^^3^
*M**_r_* = 516.69	Mo *K*α radiation, λ = 0.71073 Å
Orthorhombic, *P*2_1_2_1_2_1_	Cell parameters from 125 reflections
*a* = 9.8990 (13) Å	θ = 4.7–20.3°
*b* = 10.1030 (16) Å	µ = 0.08 mm^−^^1^
*c* = 28.961 (6) Å	*T* = 173 K
*V* = 2896.4 (8) Å^3^	Prism, colourless
*Z* = 4	0.50 × 0.30 × 0.10 mm
*F*(000) = 1128	

### Data collection

**Table:**

Bruker–Nonius KappaCCD diffractometer	5983 independent reflections
Radiation source: normal-focus sealed tube	3810 reflections with *I* > 2σ(*I*)
Graphite monochromator	*R*_int_ = 0.060
Detector resolution: 9 pixels mm^-1^	θ_max_ = 27.5°, θ_min_ = 3.2°
CCD rotation images, thick slices scans	*h* = −12→12
Absorption correction: multi-scan (*SADABS*; Bruker, 2001)	*k* = −13→13
*T*_min_ = 0.949, *T*_max_ = 0.980	*l* = −37→34
14033 measured reflections	

### Refinement

**Table:**

Refinement on *F*^2^	Secondary atom site location: difference Fourier map
Least-squares matrix: full	Hydrogen site location: mixed
*R*[*F*^2^ > 2σ(*F*^2^)] = 0.058	H atoms treated by a mixture of independent and constrained refinement
*wR*(*F*^2^) = 0.121	*w* = 1/[σ^2^(*F*_o_^2^) + (0.0514*P*)^2^ + 0.1235*P*] where *P* = (*F*_o_^2^ + 2*F*_c_^2^)/3
*S* = 1.02	(Δ/σ)_max_ < 0.001
5983 reflections	Δρ_max_ = 0.19 e Å^−^^3^
364 parameters	Δρ_min_ = −0.19 e Å^−^^3^
9 restraints	Absolute structure: Flack *x* determined using 3518 quotients [(*I*^+^)-(*I*^-^)]/[(*I*^+^)+(*I*^-^)] (Parsons *et al.*, 2013)
Primary atom site location: structure-invariant direct methods	Absolute structure parameter: −1.6 (8)

### Special details

**Table:**

Geometry. All esds (except the esd in the dihedral angle between two l.s. planes) are estimated using the full covariance matrix. The cell esds are taken into account individually in the estimation of esds in distances, angles and torsion angles; correlations between esds in cell parameters are only used when they are defined by crystal symmetry. An approximate (isotropic) treatment of cell esds is used for estimating esds involving l.s. planes.
Refinement. Some atoms of the alkyl chain are disordered over two orientations. The two split positions were refined by applying DFIX and SAME restraints on bond lengths.

### Fractional atomic coordinates and isotropic or equivalent isotropic displacement parameters (Å^2^)

**Table:**

	*x*	*y*	*z*	*U*_iso_*/*U*_eq_	Occ. (<1)
C1	0.5882 (4)	0.0382 (4)	0.39740 (12)	0.0315 (9)	
H1A	0.578278	0.020716	0.430868	0.038*	
H1B	0.503001	0.078643	0.386368	0.038*	
C2	0.6100 (4)	−0.0926 (4)	0.37238 (11)	0.0332 (9)	
H2A	0.689079	−0.138333	0.385898	0.040*	
H2B	0.529903	−0.149765	0.376963	0.040*	
C3	0.6330 (3)	−0.0730 (4)	0.32144 (12)	0.0312 (9)	
H3	0.547768	−0.042675	0.306208	0.037*	
C4	0.7455 (4)	0.0246 (3)	0.31225 (11)	0.0292 (8)	
H4A	0.751780	0.040959	0.278617	0.035*	
H4B	0.832304	−0.014254	0.322506	0.035*	
C5	0.7231 (3)	0.1563 (3)	0.33721 (11)	0.0245 (8)	
C6	0.8402 (3)	0.2523 (4)	0.32857 (10)	0.0261 (8)	
H6	0.928232	0.207118	0.334802	0.031*	
C7	0.8297 (4)	0.3746 (4)	0.35694 (11)	0.0284 (8)	
H7	0.881140	0.449469	0.347731	0.034*	
C8	0.7527 (3)	0.3857 (3)	0.39442 (10)	0.0253 (8)	
C9	0.6743 (3)	0.2708 (4)	0.41170 (11)	0.0268 (8)	
C10	0.7045 (3)	0.1369 (4)	0.39006 (11)	0.0253 (8)	
C11	0.6195 (4)	0.2791 (4)	0.45941 (12)	0.0352 (9)	
H11	0.603 (4)	0.188 (4)	0.4759 (11)	0.042*	
C12	0.6379 (4)	0.3972 (4)	0.49048 (12)	0.0365 (10)	
H12A	0.658941	0.365861	0.522048	0.044*	
H12B	0.551775	0.446946	0.491938	0.044*	
C13	0.7498 (3)	0.4907 (3)	0.47455 (10)	0.0246 (8)	
C14	0.7382 (4)	0.5105 (3)	0.42192 (11)	0.0284 (8)	
H14	0.645043	0.544747	0.415891	0.034*	
C15	0.8356 (4)	0.6248 (4)	0.41196 (12)	0.0371 (9)	
H15A	0.806930	0.674854	0.384257	0.045*	
H15B	0.928634	0.591659	0.407143	0.045*	
C16	0.8273 (4)	0.7115 (4)	0.45544 (11)	0.0375 (9)	
H16A	0.785398	0.797861	0.447990	0.045*	
H16B	0.918915	0.727750	0.467961	0.045*	
C17	0.7402 (4)	0.6359 (3)	0.49123 (10)	0.0275 (8)	
H17	0.644573	0.664645	0.486340	0.033*	
C18	0.8863 (3)	0.4285 (4)	0.48704 (11)	0.0306 (9)	
H18A	0.890760	0.414213	0.520477	0.046*	
H18B	0.959417	0.488029	0.477586	0.046*	
H18C	0.895925	0.343564	0.471051	0.046*	
C19	0.8353 (3)	0.0858 (4)	0.41279 (11)	0.0308 (9)	
H19A	0.907044	0.151705	0.408956	0.046*	
H19B	0.862571	0.002624	0.398108	0.046*	
H19C	0.819323	0.070664	0.445769	0.046*	
C20	0.7777 (4)	0.6741 (3)	0.54075 (10)	0.0292 (9)	
H20	0.876547	0.657338	0.544856	0.035*	
C21	0.7026 (5)	0.5940 (4)	0.57697 (13)	0.0500 (12)	
H21A	0.605085	0.605209	0.572699	0.075*	
H21B	0.728257	0.624897	0.607826	0.075*	
H21C	0.726093	0.500211	0.573752	0.075*	
C22	0.7531 (4)	0.8226 (3)	0.54731 (11)	0.0347 (9)	
H22A	0.810582	0.870355	0.524839	0.042*	
H22B	0.657993	0.840976	0.538966	0.042*	
C23	0.7786 (5)	0.8819 (4)	0.59447 (12)	0.0444 (11)	
H23A	0.721681	0.835638	0.617489	0.053*	
H23B	0.874292	0.867234	0.603021	0.053*	
C24A	0.7485 (4)	1.0284 (4)	0.59630 (11)	0.0338 (9)	0.489 (10)
H24A	0.649973	1.040263	0.592132	0.041*	0.489 (10)
H24B	0.793553	1.070903	0.569625	0.041*	0.489 (10)
C25A	0.7905 (9)	1.1033 (6)	0.6400 (2)	0.032 (3)	0.489 (10)
H25A	0.891186	1.107770	0.642078	0.038*	0.489 (10)
C26A	0.7327 (11)	1.0433 (8)	0.6837 (2)	0.053 (3)	0.489 (10)
H26A	0.762730	1.094987	0.710463	0.080*	0.489 (10)
H26B	0.633834	1.044421	0.682156	0.080*	0.489 (10)
H26C	0.764281	0.951815	0.686827	0.080*	0.489 (10)
C27A	0.7307 (5)	1.2442 (4)	0.63715 (13)	0.0569 (12)	0.489 (10)
H27A	0.764570	1.288414	0.609352	0.085*	0.489 (10)
H27B	0.631960	1.238800	0.635799	0.085*	0.489 (10)
H27C	0.757736	1.294718	0.664517	0.085*	0.489 (10)
C24B	0.7485 (4)	1.0284 (4)	0.59630 (11)	0.0338 (9)	0.511 (10)
H24C	0.666532	1.044727	0.577562	0.041*	0.511 (10)
H24D	0.824045	1.075446	0.581051	0.041*	0.511 (10)
C25B	0.7268 (9)	1.0915 (8)	0.6433 (3)	0.045 (3)	0.511 (10)
H25B	0.638104	1.063287	0.656574	0.054*	0.511 (10)
C26B	0.8410 (10)	1.0565 (8)	0.6758 (3)	0.053 (3)	0.511 (10)
H26D	0.825127	1.098150	0.705880	0.080*	0.511 (10)
H26E	0.845221	0.960244	0.679577	0.080*	0.511 (10)
H26F	0.926589	1.088595	0.662989	0.080*	0.511 (10)
C27B	0.7307 (5)	1.2442 (4)	0.63715 (13)	0.0569 (12)	0.511 (10)
H27D	0.658127	1.271625	0.616239	0.085*	0.511 (10)
H27E	0.718428	1.286945	0.667230	0.085*	0.511 (10)
H27F	0.818132	1.270241	0.624124	0.085*	0.511 (10)
C28	0.5848 (4)	−0.2749 (4)	0.28055 (11)	0.0342 (9)	
C29	0.6447 (4)	−0.4026 (4)	0.26568 (15)	0.0505 (12)	
H29A	0.622844	−0.471371	0.288356	0.076*	
H29B	0.743044	−0.393341	0.263366	0.076*	
H29C	0.607876	−0.427560	0.235501	0.076*	
C30	0.9279 (4)	0.2477 (5)	0.25108 (13)	0.0432 (11)	
C31	0.9098 (5)	0.3112 (5)	0.20457 (13)	0.0621 (14)	
H31A	0.925215	0.406701	0.207154	0.093*	
H31B	0.817627	0.295117	0.193519	0.093*	
H31C	0.974573	0.273129	0.182708	0.093*	
O1	0.5995 (2)	0.2137 (2)	0.32194 (8)	0.0315 (6)	
H1O	0.603 (4)	0.228 (4)	0.2944 (12)	0.038*	
O2	0.5323 (2)	0.3002 (3)	0.42006 (9)	0.0422 (7)	
O3	0.6766 (2)	−0.1988 (2)	0.30181 (8)	0.0344 (6)	
O4	0.4689 (3)	−0.2405 (3)	0.27494 (9)	0.0496 (7)	
O5	0.8345 (2)	0.2948 (2)	0.28049 (7)	0.0348 (6)	
O6	1.0105 (3)	0.1650 (4)	0.26133 (10)	0.0613 (9)	

### Atomic displacement parameters (Å^2^)

**Table:**

	*U*^11^	*U*^22^	*U*^33^	*U*^12^	*U*^13^	*U*^23^
C1	0.0277 (19)	0.034 (2)	0.0332 (19)	−0.0058 (18)	0.0024 (16)	−0.0028 (17)
C2	0.032 (2)	0.033 (2)	0.034 (2)	−0.0058 (19)	−0.0011 (17)	−0.0019 (17)
C3	0.026 (2)	0.032 (2)	0.035 (2)	0.0013 (17)	−0.0072 (16)	−0.0072 (17)
C4	0.0267 (18)	0.035 (2)	0.0264 (18)	0.0015 (18)	−0.0013 (15)	−0.0047 (16)
C5	0.0174 (17)	0.032 (2)	0.0243 (17)	0.0050 (16)	−0.0050 (14)	0.0002 (15)
C6	0.0251 (18)	0.034 (2)	0.0190 (17)	0.0006 (17)	0.0008 (14)	−0.0001 (16)
C7	0.0250 (18)	0.033 (2)	0.0268 (18)	−0.0060 (17)	−0.0078 (15)	0.0021 (16)
C8	0.0210 (18)	0.033 (2)	0.0218 (17)	0.0045 (17)	−0.0086 (15)	0.0004 (15)
C9	0.0146 (16)	0.038 (2)	0.0276 (18)	0.0034 (17)	−0.0025 (14)	−0.0034 (17)
C10	0.0209 (18)	0.032 (2)	0.0227 (17)	−0.0027 (17)	−0.0039 (14)	−0.0008 (15)
C11	0.028 (2)	0.042 (3)	0.035 (2)	−0.0083 (19)	0.0067 (16)	−0.0087 (19)
C12	0.030 (2)	0.043 (2)	0.036 (2)	−0.0077 (19)	0.0080 (16)	−0.0075 (19)
C13	0.0193 (17)	0.032 (2)	0.0228 (17)	0.0012 (17)	−0.0012 (14)	0.0012 (15)
C14	0.0248 (18)	0.033 (2)	0.0276 (18)	0.0048 (17)	−0.0059 (15)	−0.0001 (16)
C15	0.054 (2)	0.030 (2)	0.028 (2)	−0.001 (2)	−0.0026 (18)	0.0053 (17)
C16	0.056 (2)	0.030 (2)	0.0264 (19)	−0.001 (2)	−0.0036 (18)	−0.0008 (17)
C17	0.0255 (19)	0.0276 (19)	0.0293 (18)	0.0029 (18)	−0.0049 (15)	−0.0006 (15)
C18	0.031 (2)	0.031 (2)	0.0293 (18)	0.0057 (19)	−0.0062 (16)	0.0013 (17)
C19	0.0287 (19)	0.036 (2)	0.0280 (19)	0.0028 (19)	−0.0064 (16)	0.0031 (17)
C20	0.032 (2)	0.032 (2)	0.0231 (18)	0.0006 (18)	−0.0029 (15)	−0.0012 (15)
C21	0.074 (3)	0.043 (3)	0.032 (2)	−0.007 (2)	0.009 (2)	−0.0058 (19)
C22	0.037 (2)	0.033 (2)	0.0340 (19)	0.0020 (19)	−0.0040 (18)	−0.0020 (16)
C23	0.066 (3)	0.037 (2)	0.030 (2)	0.004 (2)	−0.0018 (19)	−0.0041 (18)
C24A	0.030 (2)	0.039 (2)	0.033 (2)	−0.0053 (19)	0.0006 (17)	−0.0044 (16)
C25A	0.039 (6)	0.036 (6)	0.019 (5)	0.005 (5)	0.009 (4)	−0.007 (4)
C26A	0.074 (8)	0.054 (6)	0.032 (5)	−0.021 (6)	0.004 (5)	0.003 (4)
C27A	0.080 (4)	0.038 (3)	0.052 (2)	0.017 (3)	0.004 (2)	−0.012 (2)
C24B	0.030 (2)	0.039 (2)	0.033 (2)	−0.0053 (19)	0.0006 (17)	−0.0044 (16)
C25B	0.033 (6)	0.057 (7)	0.045 (6)	0.000 (5)	0.019 (4)	−0.013 (5)
C26B	0.058 (7)	0.058 (6)	0.042 (5)	−0.013 (5)	−0.006 (5)	−0.010 (5)
C27B	0.080 (4)	0.038 (3)	0.052 (2)	0.017 (3)	0.004 (2)	−0.012 (2)
C28	0.037 (2)	0.036 (2)	0.0290 (19)	−0.006 (2)	−0.0048 (17)	−0.0032 (18)
C29	0.048 (3)	0.044 (3)	0.059 (3)	−0.003 (2)	−0.007 (2)	−0.018 (2)
C30	0.043 (3)	0.053 (3)	0.033 (2)	−0.015 (2)	0.0098 (19)	−0.009 (2)
C31	0.077 (3)	0.082 (3)	0.027 (2)	−0.029 (3)	0.013 (2)	−0.004 (2)
O1	0.0238 (12)	0.0406 (16)	0.0301 (13)	0.0059 (12)	−0.0096 (11)	0.0015 (13)
O2	0.0173 (12)	0.0556 (18)	0.0536 (16)	0.0015 (13)	−0.0003 (12)	−0.0183 (14)
O3	0.0298 (13)	0.0327 (14)	0.0408 (14)	0.0015 (13)	−0.0065 (11)	−0.0113 (12)
O4	0.0353 (16)	0.0547 (19)	0.0588 (17)	−0.0004 (15)	−0.0166 (14)	−0.0149 (15)
O5	0.0382 (14)	0.0442 (16)	0.0220 (12)	−0.0035 (14)	0.0021 (11)	0.0003 (12)
O6	0.0466 (18)	0.086 (2)	0.0515 (19)	0.0110 (19)	0.0178 (15)	−0.0129 (18)

### Geometric parameters (Å, º)

**Table:**

C1—C2	1.522 (5)	C19—H19C	0.9800
C1—C10	1.537 (5)	C20—C21	1.519 (5)
C1—H1A	0.9900	C20—C22	1.532 (5)
C1—H1B	0.9900	C20—H20	1.0000
C2—C3	1.506 (5)	C21—H21A	0.9800
C2—H2A	0.9900	C21—H21B	0.9800
C2—H2B	0.9900	C21—H21C	0.9800
C3—O3	1.458 (4)	C22—C23	1.513 (5)
C3—C4	1.511 (5)	C22—H22A	0.9900
C3—H3	1.0000	C22—H22B	0.9900
C4—C5	1.530 (5)	C23—C24B	1.511 (5)
C4—H4A	0.9900	C23—C24A	1.511 (5)
C4—H4B	0.9900	C23—H23A	0.9900
C5—O1	1.425 (4)	C23—H23B	0.9900
C5—C6	1.532 (5)	C24A—C25A	1.531 (7)
C5—C10	1.554 (4)	C24A—H24A	0.9900
C6—O5	1.458 (4)	C24A—H24B	0.9900
C6—C7	1.487 (5)	C25A—C26A	1.516 (7)
C6—H6	1.0000	C25A—C27A	1.543 (7)
C7—C8	1.331 (4)	C25A—H25A	1.0000
C7—H7	0.9500	C26A—H26A	0.9800
C8—C9	1.482 (5)	C26A—H26B	0.9800
C8—C14	1.499 (5)	C26A—H26C	0.9800
C9—O2	1.457 (4)	C27A—H27A	0.9800
C9—C11	1.487 (5)	C27A—H27B	0.9800
C9—C10	1.521 (5)	C27A—H27C	0.9800
C10—C19	1.542 (4)	C24B—C25B	1.518 (8)
C11—O2	1.445 (4)	C24B—H24C	0.9900
C11—C12	1.506 (5)	C24B—H24D	0.9900
C11—H11	1.05 (4)	C25B—C26B	1.513 (9)
C12—C13	1.528 (5)	C25B—C27B	1.553 (9)
C12—H12A	0.9900	C25B—H25B	1.0000
C12—H12B	0.9900	C26B—H26D	0.9800
C13—C18	1.533 (5)	C26B—H26E	0.9800
C13—C14	1.542 (4)	C26B—H26F	0.9800
C13—C17	1.547 (5)	C27B—H27D	0.9800
C14—C15	1.532 (5)	C27B—H27E	0.9800
C14—H14	1.0000	C27B—H27F	0.9800
C15—C16	1.536 (5)	C28—O4	1.210 (4)
C15—H15A	0.9900	C28—O3	1.340 (4)
C15—H15B	0.9900	C28—C29	1.484 (5)
C16—C17	1.550 (5)	C29—H29A	0.9800
C16—H16A	0.9900	C29—H29B	0.9800
C16—H16B	0.9900	C29—H29C	0.9800
C17—C20	1.531 (4)	C30—O6	1.206 (5)
C17—H17	1.0000	C30—O5	1.344 (4)
C18—H18A	0.9800	C30—C31	1.503 (6)
C18—H18B	0.9800	C31—H31A	0.9800
C18—H18C	0.9800	C31—H31B	0.9800
C19—H19A	0.9800	C31—H31C	0.9800
C19—H19B	0.9800	O1—H1O	0.81 (3)
			
C2—C1—C10	113.0 (3)	C10—C19—H19A	109.5
C2—C1—H1A	109.0	C10—C19—H19B	109.5
C10—C1—H1A	109.0	H19A—C19—H19B	109.5
C2—C1—H1B	109.0	C10—C19—H19C	109.5
C10—C1—H1B	109.0	H19A—C19—H19C	109.5
H1A—C1—H1B	107.8	H19B—C19—H19C	109.5
C3—C2—C1	112.0 (3)	C21—C20—C17	113.2 (3)
C3—C2—H2A	109.2	C21—C20—C22	111.0 (3)
C1—C2—H2A	109.2	C17—C20—C22	108.9 (3)
C3—C2—H2B	109.2	C21—C20—H20	107.8
C1—C2—H2B	109.2	C17—C20—H20	107.8
H2A—C2—H2B	107.9	C22—C20—H20	107.8
O3—C3—C2	108.2 (3)	C20—C21—H21A	109.5
O3—C3—C4	106.4 (3)	C20—C21—H21B	109.5
C2—C3—C4	111.7 (3)	H21A—C21—H21B	109.5
O3—C3—H3	110.2	C20—C21—H21C	109.5
C2—C3—H3	110.2	H21A—C21—H21C	109.5
C4—C3—H3	110.2	H21B—C21—H21C	109.5
C3—C4—C5	112.2 (3)	C23—C22—C20	118.3 (3)
C3—C4—H4A	109.2	C23—C22—H22A	107.7
C5—C4—H4A	109.2	C20—C22—H22A	107.7
C3—C4—H4B	109.2	C23—C22—H22B	107.7
C5—C4—H4B	109.2	C20—C22—H22B	107.7
H4A—C4—H4B	107.9	H22A—C22—H22B	107.1
O1—C5—C4	109.4 (3)	C24B—C23—C22	112.8 (3)
O1—C5—C6	110.0 (3)	C24A—C23—C22	112.8 (3)
C4—C5—C6	111.3 (3)	C24A—C23—H23A	109.0
O1—C5—C10	104.8 (2)	C22—C23—H23A	109.0
C4—C5—C10	111.9 (3)	C24A—C23—H23B	109.0
C6—C5—C10	109.3 (2)	C22—C23—H23B	109.0
O5—C6—C7	106.3 (3)	H23A—C23—H23B	107.8
O5—C6—C5	108.2 (2)	C23—C24A—C25A	117.3 (4)
C7—C6—C5	112.5 (3)	C23—C24A—H24A	108.0
O5—C6—H6	109.9	C25A—C24A—H24A	108.0
C7—C6—H6	109.9	C23—C24A—H24B	108.0
C5—C6—H6	109.9	C25A—C24A—H24B	108.0
C8—C7—C6	124.1 (3)	H24A—C24A—H24B	107.2
C8—C7—H7	118.0	C26A—C25A—C24A	113.0 (6)
C6—C7—H7	118.0	C26A—C25A—C27A	105.6 (6)
C7—C8—C9	120.6 (3)	C24A—C25A—C27A	107.9 (5)
C7—C8—C14	124.0 (3)	C26A—C25A—H25A	110.1
C9—C8—C14	115.4 (3)	C24A—C25A—H25A	110.1
O2—C9—C8	113.7 (3)	C27A—C25A—H25A	110.1
O2—C9—C11	58.8 (2)	C25A—C26A—H26A	109.5
C8—C9—C11	117.4 (3)	C25A—C26A—H26B	109.5
O2—C9—C10	116.0 (3)	H26A—C26A—H26B	109.5
C8—C9—C10	117.0 (3)	C25A—C26A—H26C	109.5
C11—C9—C10	120.3 (3)	H26A—C26A—H26C	109.5
C9—C10—C1	111.9 (3)	H26B—C26A—H26C	109.5
C9—C10—C19	106.6 (3)	C25A—C27A—H27A	109.5
C1—C10—C19	110.7 (3)	C25A—C27A—H27B	109.5
C9—C10—C5	108.5 (3)	H27A—C27A—H27B	109.5
C1—C10—C5	107.9 (3)	C25A—C27A—H27C	109.5
C19—C10—C5	111.3 (3)	H27A—C27A—H27C	109.5
O2—C11—C9	59.6 (2)	H27B—C27A—H27C	109.5
O2—C11—C12	115.2 (3)	C23—C24B—C25B	118.1 (4)
C9—C11—C12	123.8 (3)	C23—C24B—H24C	107.8
O2—C11—H11	113 (2)	C25B—C24B—H24C	107.8
C9—C11—H11	115.4 (19)	C23—C24B—H24D	107.8
C12—C11—H11	116.3 (19)	C25B—C24B—H24D	107.8
C11—C12—C13	113.4 (3)	H24C—C24B—H24D	107.1
C11—C12—H12A	108.9	C26B—C25B—C24B	110.7 (6)
C13—C12—H12A	108.9	C26B—C25B—C27B	106.5 (6)
C11—C12—H12B	108.9	C24B—C25B—C27B	108.1 (5)
C13—C12—H12B	108.9	C26B—C25B—H25B	110.5
H12A—C12—H12B	107.7	C24B—C25B—H25B	110.5
C12—C13—C18	108.3 (3)	C27B—C25B—H25B	110.5
C12—C13—C14	108.9 (3)	C25B—C26B—H26D	109.5
C18—C13—C14	110.6 (3)	C25B—C26B—H26E	109.5
C12—C13—C17	116.6 (3)	H26D—C26B—H26E	109.5
C18—C13—C17	111.7 (3)	C25B—C26B—H26F	109.5
C14—C13—C17	100.4 (3)	H26D—C26B—H26F	109.5
C8—C14—C15	118.3 (3)	H26E—C26B—H26F	109.5
C8—C14—C13	114.2 (3)	C25B—C27B—H27D	109.5
C15—C14—C13	103.7 (3)	C25B—C27B—H27E	109.5
C8—C14—H14	106.7	H27D—C27B—H27E	109.5
C15—C14—H14	106.7	C25B—C27B—H27F	109.5
C13—C14—H14	106.7	H27D—C27B—H27F	109.5
C14—C15—C16	104.0 (3)	H27E—C27B—H27F	109.5
C14—C15—H15A	111.0	O4—C28—O3	122.7 (4)
C16—C15—H15A	111.0	O4—C28—C29	126.2 (3)
C14—C15—H15B	111.0	O3—C28—C29	111.2 (3)
C16—C15—H15B	111.0	C28—C29—H29A	109.5
H15A—C15—H15B	109.0	C28—C29—H29B	109.5
C15—C16—C17	107.3 (3)	H29A—C29—H29B	109.5
C15—C16—H16A	110.3	C28—C29—H29C	109.5
C17—C16—H16A	110.3	H29A—C29—H29C	109.5
C15—C16—H16B	110.3	H29B—C29—H29C	109.5
C17—C16—H16B	110.3	O6—C30—O5	123.8 (4)
H16A—C16—H16B	108.5	O6—C30—C31	126.7 (4)
C20—C17—C13	121.1 (3)	O5—C30—C31	109.5 (4)
C20—C17—C16	111.6 (3)	C30—C31—H31A	109.5
C13—C17—C16	102.9 (3)	C30—C31—H31B	109.5
C20—C17—H17	106.8	H31A—C31—H31B	109.5
C13—C17—H17	106.8	C30—C31—H31C	109.5
C16—C17—H17	106.8	H31A—C31—H31C	109.5
C13—C18—H18A	109.5	H31B—C31—H31C	109.5
C13—C18—H18B	109.5	C5—O1—H1O	110 (3)
H18A—C18—H18B	109.5	C11—O2—C9	61.6 (2)
C13—C18—H18C	109.5	C28—O3—C3	118.6 (3)
H18A—C18—H18C	109.5	C30—O5—C6	118.3 (3)
H18B—C18—H18C	109.5		
			
C10—C1—C2—C3	−55.8 (4)	C9—C11—C12—C13	−16.5 (5)
C1—C2—C3—O3	170.0 (3)	C11—C12—C13—C18	−76.6 (4)
C1—C2—C3—C4	53.2 (4)	C11—C12—C13—C14	43.8 (4)
O3—C3—C4—C5	−171.5 (3)	C11—C12—C13—C17	156.5 (3)
C2—C3—C4—C5	−53.7 (4)	C7—C8—C14—C15	−11.4 (5)
C3—C4—C5—O1	−59.9 (3)	C9—C8—C14—C15	168.5 (3)
C3—C4—C5—C6	178.4 (3)	C7—C8—C14—C13	−133.8 (3)
C3—C4—C5—C10	55.7 (4)	C9—C8—C14—C13	46.1 (4)
O1—C5—C6—O5	−51.4 (3)	C12—C13—C14—C8	−60.6 (4)
C4—C5—C6—O5	69.9 (3)	C18—C13—C14—C8	58.3 (4)
C10—C5—C6—O5	−165.9 (3)	C17—C13—C14—C8	176.4 (3)
O1—C5—C6—C7	65.7 (3)	C12—C13—C14—C15	169.3 (3)
C4—C5—C6—C7	−172.9 (3)	C18—C13—C14—C15	−71.8 (3)
C10—C5—C6—C7	−48.8 (4)	C17—C13—C14—C15	46.3 (3)
O5—C6—C7—C8	136.3 (3)	C8—C14—C15—C16	−160.6 (3)
C5—C6—C7—C8	18.0 (5)	C13—C14—C15—C16	−33.1 (4)
C6—C7—C8—C9	2.5 (5)	C14—C15—C16—C17	7.0 (4)
C6—C7—C8—C14	−177.6 (3)	C12—C13—C17—C20	76.3 (4)
C7—C8—C9—O2	−129.4 (3)	C18—C13—C17—C20	−49.0 (4)
C14—C8—C9—O2	50.7 (4)	C14—C13—C17—C20	−166.3 (3)
C7—C8—C9—C11	164.8 (3)	C12—C13—C17—C16	−158.4 (3)
C14—C8—C9—C11	−15.1 (4)	C18—C13—C17—C16	76.4 (3)
C7—C8—C9—C10	10.2 (5)	C14—C13—C17—C16	−40.9 (3)
C14—C8—C9—C10	−169.7 (3)	C15—C16—C17—C20	152.8 (3)
O2—C9—C10—C1	−21.3 (4)	C15—C16—C17—C13	21.4 (4)
C8—C9—C10—C1	−159.9 (3)	C13—C17—C20—C21	−54.0 (4)
C11—C9—C10—C1	46.2 (4)	C16—C17—C20—C21	−175.3 (3)
O2—C9—C10—C19	−142.4 (3)	C13—C17—C20—C22	−178.0 (3)
C8—C9—C10—C19	79.0 (3)	C16—C17—C20—C22	60.7 (4)
C11—C9—C10—C19	−74.9 (4)	C21—C20—C22—C23	52.5 (5)
O2—C9—C10—C5	97.7 (3)	C17—C20—C22—C23	177.8 (3)
C8—C9—C10—C5	−41.0 (4)	C20—C22—C23—C24B	−179.2 (4)
C11—C9—C10—C5	165.2 (3)	C20—C22—C23—C24A	−179.2 (4)
C2—C1—C10—C9	174.8 (3)	C22—C23—C24A—C25A	−171.0 (5)
C2—C1—C10—C19	−66.5 (4)	C23—C24A—C25A—C26A	−55.2 (8)
C2—C1—C10—C5	55.5 (4)	C23—C24A—C25A—C27A	−171.5 (5)
O1—C5—C10—C9	−58.2 (3)	C22—C23—C24B—C25B	161.2 (5)
C4—C5—C10—C9	−176.6 (3)	C23—C24B—C25B—C26B	50.8 (9)
C6—C5—C10—C9	59.6 (3)	C23—C24B—C25B—C27B	167.2 (5)
O1—C5—C10—C1	63.2 (3)	C12—C11—O2—C9	115.8 (3)
C4—C5—C10—C1	−55.2 (4)	C8—C9—O2—C11	−108.8 (3)
C6—C5—C10—C1	−179.0 (3)	C10—C9—O2—C11	111.1 (3)
O1—C5—C10—C19	−175.2 (3)	O4—C28—O3—C3	3.8 (5)
C4—C5—C10—C19	66.4 (4)	C29—C28—O3—C3	−176.0 (3)
C6—C5—C10—C19	−57.4 (4)	C2—C3—O3—C28	98.5 (3)
C8—C9—C11—O2	102.4 (3)	C4—C3—O3—C28	−141.4 (3)
C10—C9—C11—O2	−103.9 (3)	O6—C30—O5—C6	5.5 (5)
O2—C9—C11—C12	−101.7 (4)	C31—C30—O5—C6	−175.9 (3)
C8—C9—C11—C12	0.7 (5)	C7—C6—O5—C30	131.4 (3)
C10—C9—C11—C12	154.4 (4)	C5—C6—O5—C30	−107.5 (3)
O2—C11—C12—C13	−85.5 (4)		

### Hydrogen-bond geometry (Å, º)

**Table:**

*D*—H···*A*	*D*—H	H···*A*	*D*···*A*	*D*—H···*A*
C29—H29*B*···O6^i^	0.98	2.61	3.567 (6)	166
O1—H1*O*···O4^ii^	0.81 (3)	2.16 (4)	2.923 (3)	158 (4)

Symmetry codes: (i) −*x*+2, *y*−1/2, −*z*+1/2; (ii) −*x*+1, *y*+1/2, −*z*+1/2.
